# Genetic Identity and Diversity of Loggerhead Sea Turtles in the Central Mediterranean Sea

**DOI:** 10.3390/genes15121565

**Published:** 2024-12-02

**Authors:** Adriana Vella, Noel Vella

**Affiliations:** Conservation Biology Research Group, Department of Biology, University of Malta, MSD2080 Msida, Malta; noel.vella@um.edu.mt

**Keywords:** *Caretta caretta*, conservation genetics, mitochondrial DNA control region, microsatellites, genetic structure, Mediterranean Sea, Atlantic migrants

## Abstract

**Background:** The conservation of loggerhead sea turtles (*Caretta caretta*) in the central Mediterranean benefits from an in-depth understanding of its population genetic structure and diversity. **Methods:** This study, therefore, investigates *C. caretta* in Maltese waters by genetically analysing 63 specimens collected through strandings and in-water sampling, using mitochondrial DNA control region and microsatellites. Additionally, the two nests detected in Malta in 2023 were analysed for the same markers. **Results:** Mitochondrial data identified 10 haplotypes, with mixed stock analyses tracing 87.5% of the specimens to Mediterranean origins, primarily from Libyan rookeries, with contributions from Lebanon, Israel and Turkey. Three Atlantic haplotypes were identified in six specimens, with CC-A17.1 linking central Mediterranean foraging individuals to rookeries in Cape Verde. Five of these six Atlantic haplotype records were from recently sampled individuals (2022–2023), possibly indicating a recent eastward expansion of Atlantic haplotypes into the Mediterranean. Bayesian clustering (K = 2) of microsatellite data using haplotypes as priori revealed similar proportions for clusters across most specimens, except for three specimens with Atlantic haplotypes CC-A1.1 and CC-A1.3, which exhibited distinct patterns. The two nests examined here displayed Mediterranean haplotypes, with nuclear DNA matching the predominant Mediterranean profiles found in foraging individuals, suggesting that local clutches originated from Mediterranean parents. **Conclusions:** Increasing nesting activity on Maltese beaches and this archipelago’s geographical position highlight the need for ongoing genetic monitoring to track changes in genetic diversity and develop conservation strategies that support the effective protection of this species and its habitats.

## 1. Introduction

Three species of turtles are found in the Mediterranean Sea: two nesting Cheloniidae species and the migratory *Dermochelys coriacea* [1,2,3], with the loggerhead sea turtle, *Caretta caretta* (Linnaeus, 1758), being the most common in the region [4,5]. According to the IUCN, this species is globally classified as *Vulnerable* with a decreasing population trend [6]. The North East Atlantic subpopulation is classified as *Endangered* [7], while the subpopulations in the Northwest Atlantic and the Mediterranean are considered as *Least Concern*, with the Mediterranean Regional Management Unit (RMU) regarded as conservation-dependent [8,9]. The species is protected under several international agreements [10,11,12,13,14], while regional fisheries management organisations provide recommendations to fishery managers on mitigating accidental catches of turtles [15,16].

As a highly migratory species, *C. caretta* exhibits a complex life history. After hatching, juveniles enter an oceanic phase, drifting with currents for several years and often covering vast areas, sometimes crossing entire oceans. As they grow into sub-adults and adults, they begin long-distance migrations, displaying philopatric behaviour by returning to their natal region [17]. Once fully mature, adults typically migrate between feeding and breeding grounds every few years [18]. In the Mediterranean, one key migratory corridor spans from Tunisia to Greece and Turkey, passing through the Sicilian channel near Malta [19], which is the area studied here. This migratory behaviour influences the population structure of *C. caretta*, with juvenile populations exhibiting little to no genetic structure, but as the turtles mature, genetically distinct populations become evident [20,21].

In the Mediterranean Sea, native *C. caretta* frequently shares foraging areas with juveniles of Atlantic origin [22,23,24,25]. These Atlantic juveniles are carried into the Mediterranean by prevailing currents through the Strait of Gibraltar [26], where they remain until they grow large enough to actively swim against these currents and migrate back to the Atlantic. Genetic data confirm this movement, as haplotypes typically associated with Atlantic rookeries are more prevalent in the western Mediterranean, particularly in the Alboran Sea [27,28,29,30]. The proportion of *C. caretta* of Atlantic origin in the Mediterranean foraging grounds declines as carapace length increases as subadults migrate back to their natal regions [29]. The return migration of Atlantic sub-adults from the Mediterranean Sea is influenced by climatic events, with unfavourable conditions increasing the abundance of *C. caretta* in the Gulf of Cádiz and the Alboran Sea [31].

Although juveniles from Atlantic rookeries may spend years foraging in the Mediterranean, adults will ultimately return to their natal beaches in the Atlantic to reproduce. Satellite tagging data show that some sub-adults migrate from the Mediterranean as far as the Caribbean Sea [17,24,32]. This behaviour limits interbreeding between Atlantic and Mediterranean populations [33], maintaining distinct genetic lineages [22] and resulting in separate Regional Management Units (RMUs) [34].

Mitochondrial DNA (mtDNA) data from Mediterranean rookeries display a unique set of haplotypes largely distinct from those in the Atlantic. The limited overlap of haplotypes between these populations suggests that the Mediterranean experienced few colonisation events through the Atlantic Ocean, likely during the Pleistocene [35,36], with the founding females carrying only a subset of haplotypes. These haplotypes would have included the commonly shared haplotype CC-A2.1 [35,37], but over time, the Mediterranean population expanded and evolved new haplotypes that are now exclusive to Mediterranean rookeries [23,35,38]. Due to female philopatry, some of these new haplotypes remain confined to specific beaches within the Mediterranean Sea [29,35]. This small-scale variation has led to the subdivision of the Mediterranean RMU into smaller Management Units (MUs), each associated with established rookeries along various beaches in the central and eastern Mediterranean [1,39].

Currently, no MUs are designated for the western Mediterranean, as there are no major rookeries, and nesting events in the region are often sporadic. Recent studies, however, have documented an increase in nesting records, a trend that may partly be due to increased awareness and citizen science efforts but also due to an actual upward trend in nesting activity, possibly linked to climate change. Nesting hotspots have been identified along beaches in southwest Italy, southeast Sardinia, and northwest Tunisia [40,41]. Genetic analyses of nests in this region revealed the presence of haplotypes CC-A1.1 and CC-A9.1, typically associated with Atlantic nests, in two out of 18 nests in Carreras et al. [28] and one out of 11 nests in Luna-Ortiz et al. [33]. Although these sample sizes are small, they suggest that the western Mediterranean could potentially be experiencing colonisation by new settlers adapting to changing environmental conditions [42]. This adaptation could reflect behavioural plasticity in response to climate change [33,43,44]. Another possible influence may include Earth’s shifting magnetic field, affecting the natal homing and geomagnetic spatial orientation during navigation imprinted at birth [45].

Further genetic characterisation of nests in the western Mediterranean, as well as other recently colonised beaches, may help clarify the influence of sporadic nesting events on the genetic boundaries shaped by philopatry [28] between the Mediterranean and the Atlantic Ocean and between Mediterranean MUs.

In the central and eastern Mediterranean, ecological models project minimal phenological shifts in post-nesting migratory corridors between major nesting sites and foraging grounds [19,46]. However, Petsas et al. [46] concluded that parts of the corridor between the foraging grounds in the Gulf of Gabes, Tunisia, to nesting grounds in Greece and Turkey may shift eastwards, potentially increasing the selection of Maltese waters.

To ensure the long-term survival of *C. caretta* populations in the Mediterranean Sea, a unified approach involving comprehensive scientific data collection and shared conservation strategies is essential [47]. This approach requires standardised methods that enable international collaboration and effective data sharing on the species [48,49]. Key research needs include determining the origin of sea turtle strandings, as well as the identification of juveniles, sub-adults, and adults found at sea, along with the genetic characterisation of foraging populations. This information is crucial for delineating population boundaries in marine habitats by identifying the genetic profile of key nesting sites and determining the origins of turtles in foraging areas. Establishing the genetic origins of a population relies on baseline information about the species’ population structure and the use of genetic tools, such as mtDNA and nuclear DNA (nDNA) markers, to distinguish defined populations [50,51,52]. Applying genetic tools to study flagship and vulnerable species in Maltese waters provides an opportunity to enhance our understanding of the central Mediterranean’s significance for the diverse ecological and life-history needs of migratory species [53,54,55].

This study analysed the genetic diversity of *C. caretta* within Maltese waters in the central Mediterranean, a region that connects the nesting sites in Calabria, Italy [56], and nesting sites along Greek and Turkish coasts [19,46], with foraging grounds in Tunisian and central Mediterranean waters. Malta has a number of MPAs specifically designed for the conservation of *C. caretta* [57], and over the past decade, there has been a noticeable increase in nesting events along Maltese shores [58]. To investigate the genetic diversity and origin of *C. caretta* individuals frequenting Maltese waters, we utilised the highly variable mtDNA control region [59,60], which is widely recognised as a standard sequence for most genetic studies on the species [20,21,23,24,37,51,61,62]. Additionally, we included 25 microsatellite loci [52,63,64,65,66,67,68], which are the same set used in an earlier genetic study on *C. caretta* nests in the same area [58]. The main objectives of this research were (1) to characterise the genetic diversity of *C. caretta* individuals from Maltese waters by analysing the mtDNA control region and microsatellite loci; (2) to infer the origin of these sea turtles through mixed stock analysis and the Bayesian algorithm, STRUCTURE; and (3) to examine the genetic relationships between different nests using computational sibship and parentage analysis. Together, these analyses aimed to improve our knowledge of the genetic composition and connectivity of *C. caretta* individuals frequenting Maltese waters.

## 2. Materials and Methods

### 2.1. Study Area and Background Information

**Figure 1:**
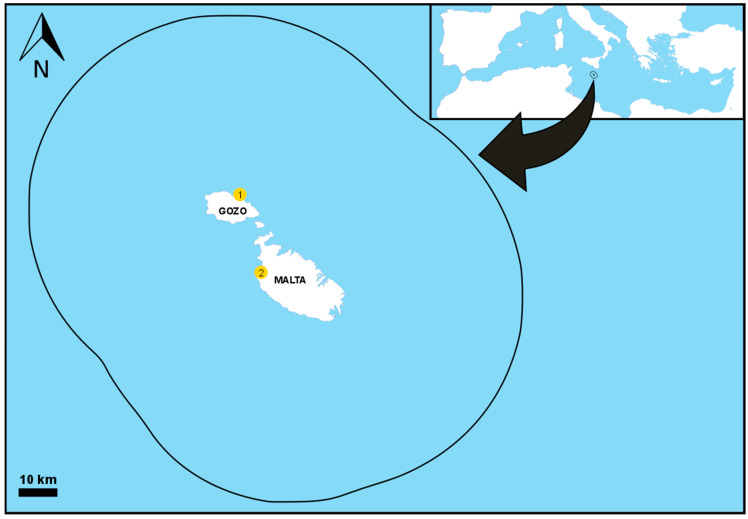
Map showing the Maltese Fisheries Management Zone (circle), where sampling of strandings and in-water sampling was conducted, including the location of the analysed nesting sites: 1—Ramla Bay, Gozo; 2—Ġnejna Bay, Malta.

In this study, we sampled 63 juvenile and sub-adult *C. caretta* from the Maltese Fisheries Management Zone (FMZ), which is 25 nautical miles around the Maltese islands (Figure 1). Tissue samples used for this study were collected during three sampling periods: from strandings in 2008–2009 (n = 14) and from free-living foraging individuals in 2014–2018 (n = 15) and 2022–2023 (n = 34). Sampling was carried out in accordance with the national Environment Resource Authority (ERA) permits issued to AV. Additionally, two *C. caretta* nests laid in the summer of 2023 on two different beaches, Ramla Bay, Gozo (six dead specimens), and Ġnejna Bay, Malta (one dead specimen) (Figure 1), were genetically studied through tissue samples from dead hatchlings and embryos collected from post-hatch nest excavations. The latter were collected by the local authority ERA and handed over for tissue sampling and scientific investigation, as per ERA permits issued to AV.

### 2.2. Sample Collection, DNA Extraction, PCR Amplification and Sequencing

*Caretta caretta* specimens were identified and measured following Rene Marquez [69]. The curved carapace length (CCL) of the sampled turtles in this study ranged from 20.0 cm to 69.0 cm, with a mean of 40.4 cm (SD ± 13.0 cm). The largest individual, with a CCL of 69.0 cm, is close to the average minimum size for nesting females in the Mediterranean Sea [70,71,72], indicating that sampled individuals ranged between juveniles and sub-adults. In the central Mediterranean, turtles typically attain full maturity at a curved carapace length of over 75 cm [73].

Skin tissue samples were collected from both stranded and free-living individuals using 3 mm biopsy punches, following established procedures [74,75]. This tissue collection followed research permits from the Environment Protection Authority (ERA) in Malta and institutional UM Research Ethics approval. Biopsies collected were stored in 100% ethanol. The total genomic DNA was subsequently extracted from tissue samples using the GF-1 Tissue DNA Extraction Kit (Vivantis Technologies, Selangor, Malaysia) according to the manufacturer’s instructions. The concentration of the purified DNA was measured using a Qubit fluorometer (ThermoFisher Scientific, Waltham, MA, USA).

All specimens collected were analysed for their mtDNA CR using LCM15382 and H950 primers [76], following the amplification protocol described by Shamblin et al. [49]. PCR products were checked for successful amplification on 1.5% agarose gel, and PCR product concentration was estimated using Qubit (ThermoFisher Scientific, Waltham, MA, USA). Purified PCR products were sequenced with the respective forward and reverse primers using the ABI3730XL sequencer (ThermoFisher Scientific, Waltham, MA, USA).

All specimens were analysed for 25 microsatellite loci, matching the selection of loci used for another study on *C. caretta* from Maltese waters [58]. These markers included 12 dinucleotide microsatellite loci [52,63,64,65,66] and 13 tetranucleotide microsatellite loci [67,68]. The primers were all tagged with M13 tails, fluorescently labelled and amplified according to Vella and Vella [58]. PCR products were size-scored using ABI3730XL. To estimate the error rate, 15% of the specimens were randomly selected and analysed twice for each microsatellite locus.

### 2.3. Mitochondrial DNA Statistical Analyses

Mitochondrial DNA sequences were manually trimmed, and the complementary sequences for each specimen were assembled using Geneious R10 [77]. DnaSP v6 [78] was utilised to identify and categorise specimens based on their haplotypes. A haplotype rarefaction curve was created using Analytic Rarefaction v.1.3 (http://stratigrafia.org/software/index.html, accessed on 8 August 2024) to illustrate the level of saturation concerning the number of specimens sampled compared with the detected haplotypes. Curves demonstrating quick saturation and convergence of the confidence intervals suggest that sufficient sampling was performed to capture the existing genetic variation [79].

All genetic sequences were compared to publicly available sequences using BLASTn (https://blast.ncbi.nlm.nih.gov/ accessed: 5 August 2023) [80,81] to identify the mtDNA haplotype. Haplotype designations for the sequences obtained were determined according to the naming conventions set forth by the Archie Carr Center for Sea Turtle Research database [48]. Genetic diversity indices were calculated using Arlequin v3.5 [82]. To illustrate the genetic relationships between the identified haplotypes, a minimum-spanning haplotype network was constructed using TCS v1.21 [83].

Pairwise F_ST_ and φ_ST_ values between sampling years were calculated using 1 × 10^5^ permutations via Arlequin v3.5 [82], utilising the Tamura-Nei 1993 model [84] as the model of best fit for DNA evolution as selected via the jModelTest 2.1.7 [85]. A mixed stock analysis was conducted to estimate the geographic origins of the specimens, using haplotype frequencies from Atlantic and Mediterranean rookeries as baseline data [49,86], following the approach applied by Tolve et al. [38] and Loisier et al. [23]. This analysis was performed with the MIXSTOCK package v0.9.9 [87] in RStudio v2024.04.2.

We also compared the haplotypes identified in this study to those found in other studies on foraging individuals. For this comparison, we used absolute frequencies from studies that employed long mtDNA control region sequences (>800 bp), with sample sizes exceeding 50 individuals to ensure adequate coverage of haplotype diversity, and excluded hatchling data. Data were obtained for the Mediterranean French coast [23], the Adriatic Sea [38], Turkey [61] and the North Atlantic [51]. Pairwise F_ST_ and φ_ST_ values between these geographical locations were calculated using Arlequin v3.5 [82] utilising the Tamura model [88].

### 2.4. Microsatellites Analyses

Microsatellite allele sizes were scored using Geneious R10 [77], and specimens failing to amplify for more than four loci were excluded from further analyses. Genotypes were validated with Micro-Checker v2.2.3 [89]. Pairwise linkage disequilibrium was tested in Arlequin v3.5 [82] using 10,000 permutations (burn-in 10%) to ensure loci independence. Arlequin v3.5 was also used to determine the number of alleles, observed heterozygosity (H_o_), expected heterozygosity (H_E_) and deviations from the Hardy–Weinberg equilibrium at each locus, with significance levels adjusted using Bonferroni correction. Polymorphic information content (PIC) was estimated with Cervus v3.0.7 [90]. F_ST_ values were calculated in Arlequin v3.5 [82] to assess genetic differentiation by sampling period.

Bayesian clustering analysis in STRUCTURE v2.3.4 [91] was used to estimate the likelihood that a specimen belongs to an inferred cluster under a non-admixture model with correlated allele frequencies suitable for populations with minimal mixing [92,93,94]. Mitochondrial DNA haplotypes were used as priori to assist clustering [92,94]. Analyses used a burn-in of 4 × 10^5^ and 2 × 10^6^ MCMC replications for K = 1 to K = 7. Each run was replicated 20 times, and the outputs were analysed for the best value of K using StructureSelector [95], adopting various models for data interpretation [96,97,98]. Outputs were then visualised using CLUMPAK [99] within StructureSelector through a LargeKGreedy algorithm, random input order and 2000 repeats.

Genetic differentiation was further analysed with principal component analysis (PCA) followed by discriminant analysis of principal components (DAPCs) in adegenet package v2.1.10 [100] in RStudio v2024.04.2. DAPC segregates genetic variation to enhance distinctions between groups while minimising differences within each group. Cross-validation using the function xvalDapc was conducted to identify the optimal number of principal components (PCs) for the analysis. A density plot of DF1 scores was generated to visualise group separation.

Analysis of molecular variance (AMOVA) was conducted for various combinations of specimens using haplotypes as priori through Arlequin v3.5 [82]. BOTTLENECK v1.2. [101,102] was used to detect potential recent population reductions using the Wilcoxon signed-rank test. Heterozygosity excess was tested under the infinite allele model (IAM), the stepwise mutation model (SMM) and the two-phase model (10% IAM and 90% SMM) using 1000 iterations. COLONY v2.0.6.8 [103,104] was used to determine whether there were any full-sibling or half-sibling relationships between the analysed specimens.

### 2.5. Nests’ Hatchling Remains Analyses

The data on dead hatchlings were analysed using COLONY v2.0.6.8 [103] to assign sibship and parentage among specimens using likelihood-based methods through multi-locus genotype data with an error rate of less than 1%. Additionally, data by Vella and Vella [58] were included to enhance the detection of females returning to nests in Malta across different seasons. In a separate run, the same STRUCTURE parameters used for in-water and stranded specimens were applied to one representative specimen from each nest, aiming to assess the likelihood that hatchlings belong to the inferred genetic clusters.

## 3. Results

### 3.1. In-Water and Stranded Specimens

#### 3.1.1. Mitochondrial DNA

The mtDNA region of the 63 specimens analysed ranged from 857 bp to 859 bp, with most of this sequence representing the control region [105]. Ten haplotypes were identified, with a haplotype diversity of 0.525 (SD ± 0.074), a nucleotide diversity of 0.0090 (SD ± 0.0047) and a mean pairwise difference of 7.71 (SD ± 3.64). In total, 47 polymorphic sites were identified, including nine indels and 38 transitions, of which two were singletons and the rest were parsimony-informative. Although a larger sample size might reveal additional rare haplotypes, rarefaction analysis generated a curve that nearly plateaued with converging 95% confidence intervals (Figure S1), suggesting that the sampling strategy effectively captured the majority of haplotypes present in the region.

**Figure 2:**
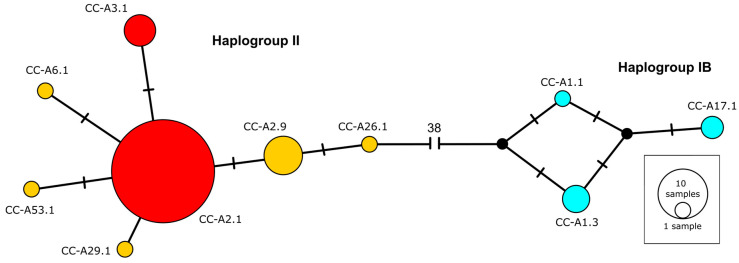
Haplotype network for the mtDNA control region sequences analysed from 63 specimens from Maltese waters. Circle sizes are proportional to sample frequencies. Circles: black indicates undetected haplotypes; yellow indicates haplotypes exclusive to the Mediterranean Sea; blue indicates haplotypes exclusive to the Atlantic Ocean; red indicates haplotypes of mixed origin. Haplogroup II (**left**) and Haplogroup IB (**right**) are labelled according to Shamblin et al. [49].

The most common haplotype identified was CC-A2.1 (Table 1; Figure 2), found in 43 specimens (68.2%), followed by CC-A29.1 (9.5%), CC-A3.1 (6.3), CC-A1.3 (4.8%) and CC-A17.1 (3.2%). Haplotypes CC-A1.1, CC-A26.1, CC-A29.1, CC-A53.1 and CC-A6.1 were each found in one specimen. Six specimens had haplotypes (CC-A1.1, CC-A1.3, CC-A17.1) clustering into Haplogroup IB [49], associated with Atlantic rookeries. The rest of the specimens clustered into Haplogroup II, including 10 specimens with haplotypes exclusive to Mediterranean rookeries (CC-A2.9, CC-A26.1, CC-A29.1, CC-A53.1, CC-A6.1). The majority of the specimens (74.6%) had haplotypes that are shared between Mediterranean and Atlantic rookeries (CC-A2.1, CC-A3.1) [23,29] (Table 1).

F_st_ and φ_ST_ analyses indicated no significant differences between the samples based on sampling year (Table 2). However, samples collected in 2008–2009 exhibited a lower haplotype and nucleotide diversity compared with those collected in 2014–2018, with the highest level of genetic diversity being noted in samples from 2022–2023. Additionally, the Atlantic haplotypes CC-A1.1, CC-A1.3 and CC-A17.1 became more frequent in recent sampling periods (Table 1). While these trends may be associated with increased sample sizes in recent years, they could also suggest potential temporal shifts in the population structure of *C. caretta*.

**Figure 3:**
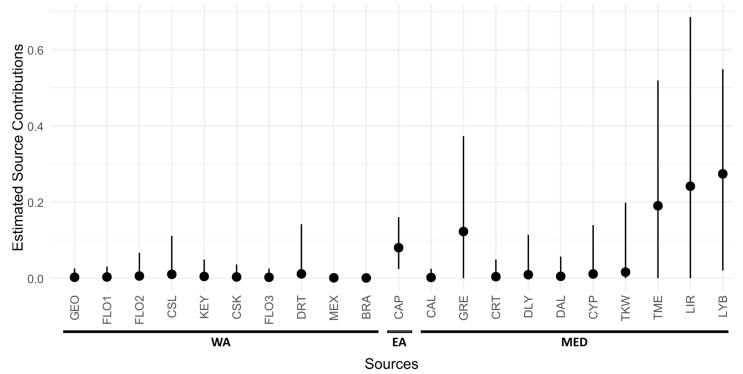
Mixed stock analysis displaying the contribution proportions and 95% confidence intervals for 21 groups of rookeries from the Atlantic (WA—west Atlantic; EA—east Atlantic) and Mediterranean Sea (MED) to the *Caretta caretta* population in Maltese waters. Abbreviations as indicated in Table 1.

The mixed stock analysis indicated that 87.5% were of Mediterranean origin, with the remaining 12.5% of Atlantic origin (4.5% from the western Atlantic and 8.0% from Cape Verde) (Figure 3). Among the Mediterranean contributions, the highest percentage was from Libya (LYB: 27.4%), with other high contributions from eastern Mediterranean coasts, including Turkey (TME: 19.0%), Lebanon, Israel (LIR: 24.1%) and Greece (GRE: 12.3%). Contributions from other Mediterranean regions were lower than 2% (Figure 3). The contributions of all rookeries, except for those of Libya and Cape Verde, have confidence intervals that overlap with zero, an observation noted in other similar studies using the same marker [23,38,51,61]. Therefore, although widely used, results are often limited in precision due to low resolution and the widespread presence of haplotypes CC-A2.1 and CC-A3.1 across rookeries. Despite these limitations, the mtDNA control region remains the main marker for linking foraging populations to nesting sites. The adoption of more sensitive markers to global rookeries, such as complete mitogenomes and genomic studies [33,60], may improve the accuracy of mixed stock analyses in the future.

#### 3.1.2. Microsatellites

We genotyped 61 out of the 63 analysed specimens. Two specimens with haplotype CC-A2.1 were excluded from further analysis as they failed to amplify for more than four loci. The number of alleles per locus ranged between four alleles (CcP5C11) to 16 alleles (Cc7B07), with a mean of 10.32 SD ± 3.67 alleles per locus (dinucleotide loci: 8.33 SD ± 2.60; tetranucleotide loci: 12.15 SD ± 3.63) (Table 3). Micro-checker analysis [89] showed no evidence of scoring errors due to stuttering, no large allele dropout and no indication of null alleles. All replicate genotypes were identical. Linkage disequilibrium (LD) was exhibited by 9% of the loci pairs at *p* < 0.05; however, no LD was observed after Bonferroni correction. Therefore, all loci were used for subsequent analyses.

Observed heterozygosity (H_o_) ranged from 0.426 (Cc-2) to 0.967 (Cc5H07), with the mean H_o_ being 0.741 SD ± 0.149 (dinucleotide loci: 0.662 SD ± 0.142; tetranucleotide loci: 0.814 SD ± 0.166). Three loci (CcP1F09, Cc-28 and Cc-30) departed from the Hardy–Weinberg expectations at *p* < 0.05; however, no significant deviations were noted after Bonferroni correction. The PIC ranged from 0.407 (Cc-2) to 0.894 (Cc1G02), with an average PIC of 0.741 SD ± 0.135 (dinucleotide loci: 0.660 SD ± 0.124; tetranucleotide loci: 0.821 SD ± 0.093) (Table 3).

F_ST_ values for microsatellite analyses between different years were generally low (Table 2), with no significant differences after Bonferroni correction. Analysis through COLONY indicated that none of the foraging specimens studied here were identified as siblings or half-siblings, suggesting a lack of close familial relationships within the sampled population. This finding implies a broad genetic diversity among the turtles analysed, with specimens likely originating from different parental pairs.

**Figure 4:**
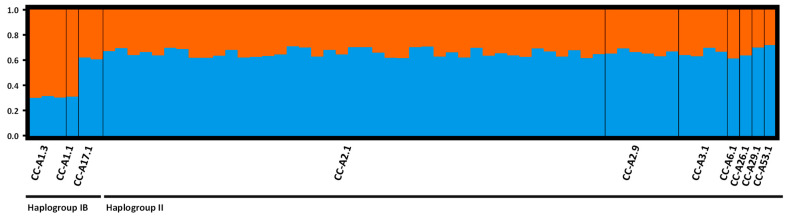
Bar plot illustrating the genetic structure of 61 specimens analysed using microsatellites, with K = 2 clusters identified under a no-admixture, frequency-correlated model. The mtDNA control region haplotypes were used as prior information. Each bar represents a specimen, with the proportion of each colour indicating the assignment probability to each of the two clusters. Haplotypes associated to Atlantic rookeries: CC-A1.3; CC-A1.1; CC-A17.1; Haplotypes associated to Mediterranean rookeries: CC-A2.9; CC-A6.1; CC-A26.1; CC-A29.1; CC-A53.1; Haplotypes associated to Atlantic and Mediterranean rookeries: CC-A2.1; CC-A3.1.

Bayesian analyses using Structure [91] identified an optimal number of clusters at K = 2 after filtering out spurious results with StructureSelector [95]. This finding was corroborated using the Puechmaille method and further supported using the Evanno method [96,97,98]. CLUMPAK analysis [99] showed that specimens carrying haplotypes CC-A1.1 and CC-A1.3 had the lowest assignment values to the second cluster (~30%), while for the other specimens, the assignment values to the second cluster exceeded 60% (Figure 4). Notably, the two specimens with haplotype CC-A17.1, despite belonging to Haplogroup IB, showed nDNA profiles similar to those of Haplogroup II (Figure 4). Genetic divergence was also observed in DAPC analysis, which differentiated specimens with haplotypes CC-A1.1 and CC-A1.3 from the rest. As shown in the density plot of Discriminant Function 1 (DF1) (Figure S2), the specimens carrying CC-A1.1 and CC-A1.3 exhibited a distinct peak with minimal overlap, indicating a genetic distinction between the groups.

AMOVA analyses (Table 4) using haplotypes as priors showed that the comparison between Haplogroup IB and Haplogroup II accounted for 3.58% of the variation (F_ST_ = 0.039, *p* = 0.0123). In contrast, the variation increased to 6.08% (F_ST_ = 0.061; *p* = 0.0122) when comparing specimens with haplotype CC-A1.3 against all other haplotypes, suggesting a small but significant nDNA differentiation between specimens having Atlantic haplotypes, namely CC-A1.1 and CC-A1.3, and the rest.

Analyses of microsatellite data using BOTTLENECK detected no significant population bottleneck events under the TPM and SMM models (Table S1), as indicated by non-significant *p*-values from the Wilcoxon signed-rank test. In contrast, the IAM model showed a significant level of heterozygosity excess. However, the IAM model is not always suitable for analysing microsatellites, especially those exhibiting a high degree of single-step mutations, as it can lead to an overestimation of heterozygosity excess [101,102]. The absence of a bottleneck was further supported by the L-shaped allele frequency distributions observed under both the TPM and SMM models (Table S1). The testing for a bottleneck assumes that recent events would have left detectable genetic signatures, as observed in other studies using similar markers [107,108].

#### 3.1.3. Mitochondrial DNA and Phylogeography

Most pairwise comparisons showed significant differences between foraging populations, with the strongest distinctions observed between the Atlantic and the Mediterranean populations (*p*-values < 0.0001). Among Mediterranean foraging areas, the closest genetic relationship to the Maltese specimens was found with those along the French coast [23], as the small genetic difference noted between the two areas was not significant when applying the Bonferroni correction (F_ST_ = 0.0169; *p*-value = 0.1161). Similarly, the φ_ST_ value for Maltese specimens compared with those from Turkey was significant at a *p*-value of 0.05 but did not remain significant after the Bonferroni correction. All other pairwise comparisons involving Maltese specimens produced φ_ST_ and F_ST_ values that indicate significant genetic differentiation after Bonferroni correction (Table 5).

### 3.2. Nests

In 2023, only two nests were identified, one on the island of Gozo and one on the island of Malta (Figure 1). Both nests yielded the same haplotype, CC-A2.1, while microsatellite data indicated that these two nests were unrelated to each other (Table S2). No parental link was found between the 2023 nests and previously analysed Maltese nests laid between 2020 and 2022 [58], indicating that two new females laid eggs on Maltese beaches (Table 6). Although multiple paternity was not detected, the ability to identify such events in the 2023 nests was limited due to the sample size available.

## 4. Discussion

### 4.1. Mitochondrial Lineages

Mitochondrial DNA analyses indicate the *C. caretta* frequenting Maltese waters belong to two lineages, Haplogroup IB (9.4%) and Haplogroup II (90.6%), which represent the main lineages found in the Atlantic Ocean and the Mediterranean Sea [49]. These proportions are similar to findings along the Mediterranean French coast [23] but differ from those in the Alboran Sea, which shows a higher presence of Haplogroup IB, and the Adriatic Sea and the eastern Mediterranean, where Haplogroup IB is less common [27,38,61]. Results also indicate an increase in haplotype diversity over time, with the lowest diversity (0.264 SD ± 0.136) observed in the early sampling period, consistent with findings by Garofalo et al. [109], and a more recent index of 0.608 SD ± 0.092. This rising diversity may be due to increased sample sizes but could also reflect an influx of new migrants from other Mediterranean regions and the Atlantic, enhancing the genetic diversity of the population around Malta.

The most common haplotype in this study was CC-A2.1 (68.3%), an ancestral haplotype to Mediterranean populations and the most frequent in Mediterranean nests (Table 6), including those from Malta [58]. This haplotype represents the majority of the analysed Mediterranean specimens [23,38,86,106,109] and is also found in Atlantic rookeries [35,49]. The second most common haplotype was CC-A2.9 (13.9%), which is exclusively Mediterranean [110] and the second most common haplotype in Libyan rookeries [106,110]. Four specimens carried CC-A3.1, a haplotype commonly found across Mediterranean rookeries [23,29,38,58,106], and is shared with Atlantic rookeries [35,49]. The remaining haplotypes from Haplogroup II (CC-A26.1, CC-A29.1, CC-A53.1 and CC-A6.1) were each represented by one specimen. These are specific to Mediterranean rookeries, with CC-A26.1 being associated with Libya, CC-A29.1 with Israel, CC-A53.1 with eastern and middle Turkey, and CC-A6.1 with Western Greece [29,106].

Mixed stock analysis using mtDNA suggests that the *C. caretta* frequenting Maltese waters are linked to multiple MUs, primarily from Libya (27.4%), Lebanon and Israel (24.1%), Turkey (DLY + DAL + TKW + TME 22.0%), and Greece (12.3%). These findings align with those by Garofalo et al. [109], who identified turtles in the central Mediterranean with major contributions from rookeries in Libya, Turkey, Greece, Israel and Lebanon. These support the migratory corridors between nesting grounds in Greece and Turkey to foraging grounds in the Gulf of Gabes, with satellite tracking showing that migration between these areas often crosses Maltese waters [25,46]. The limitations in the distribution of satellite-tracked foraging turtles are overcome by mtDNA analyses, which showed that there are contributions coming from more distant nesting sites, matching with the close genetic relationship between the rookeries in Israel, Lebanon and Libya [111].

In Maltese waters, Haplogroup IB was represented by CC-A1.3 (4.8%), CC-A17.1 (3.2%) and CC-A1.1 (1.7%). These haplotypes are associated with Atlantic nesting sites, with CC-A1.1 being found mostly in western Atlantic rookeries, such as Florida, CC-A1.3 being linked to several Atlantic beaches but primarily found in Cape Verde, and CC-A17.1 being unique to Cape Verde [49,112]. CC-A1.1 has also been detected in two nests in the western Mediterranean—in a 2006 nest at Premià de Mar, Spain [28], and in a 2017 nest at Peñiscola, Spain [33]—but was absent in larger studies covering nests in the southern, central and eastern Mediterranean [106]. Foraging individuals with CC-A1.1 and CC-A1.3 were found in the Balearic Sea, the Mediterranean French coast, the Algerian basin, the Tyrrhenian basin and Lampedusa [23,29,109], with one record of CC-A1.1 in the southern Levantine Sea [29]. Our results provide some of the most easterly records of CC-A1.3 and the first detection of CC-A17.1 in the Mediterranean, suggesting potential range shifts for these Atlantic haplotypes. Although sample sizes for Atlantic haplotypes remain small, their increased prevalence in recent sampling years may indicate an eastward shift in distribution by Atlantic-origin turtles. This highlights the importance of monitoring genetic patterns to understand shifts in habitat utilisation across regions.

Mixed stock analysis indicated an Atlantic contribution of approximately 12.5%, similar to the 11% noted along the Mediterranean French coast [23] but lower than the 60% reported by Clusa et al. [29] for the juveniles in the Algerian basin. Atlantic contributions to the Mediterranean generally range from 5% to 20%, with higher proportions along the southern coasts of the western Mediterranean [29]. Our findings identify Cape Verde as the primary Atlantic source (8.0%), with smaller contributions from the western Atlantic, contrasting with Loisier et al. [23], who identified Florida and Bahamas rookeries as the main Atlantic contributors. Our results also differ from those by Garofalo et al. [109], who, using shorter mtDNA sequences, attributed the Atlantic origin of central Mediterranean *C. caretta* to rookeries in the Dry Tortugas and Yucatan, Mexico.

These differences may be explained by the higher presence of CC-A17.1 and CC-A1.3 in our study, both common in Cape Verde rookeries, with CC-A17.1 being exclusive to rookeries in that region [21,36,51,112]. Baltazar-Soares et al. [36], suggested that Haplogroup II migrated along South African and western African coasts, passing near Cape Verde, before colonising the Mediterranean Sea. Later, the Mediterranean populations may have used Cape Verde as a stepping stone for the colonisation of the Atlantic with Haplogroup II. Although the contribution of Cape Verde to the Mediterranean is commonly overlooked, possibly because migrants carry common haplotypes not unique to the region, the presence of Haplogroup I specimens with CC-A17.1 suggests that the role of the eastern Atlantic in shaping the Mediterranean populations needs further investigation.

### 4.2. Biparental Markers

Microsatellite data showed minimal nuclear differentiation among specimens (Figure 4), with those carrying Haplogroup II showing similar contributions from the two clusters identified by STRUCTURE. This suggests that while females exhibit strong philopatry by returning to specific nesting beaches, male-mediated gene flow leads to genetic mixing, reducing the ability of nDNA data to distinguish the origin of closely related specimens, such as those from the same RMU [23]. Although Clusa et al. [111] challenged this by proposing that both female and male *C. caretta* exhibit philopatry, other studies on genetic structure have shown that while mitochondrial DNA reveals significant structuring based on past colonisations, microsatellite analyses fail to detect such structuring, even among colonies differentiated by maternal lineages [23,52,109,112]. This may be due to opportunistic mating by a few males over successive generations as females migrate to their respective nesting beaches. Additionally, mixed stock analysis of our data indicates that the largest contributions are from Lebanon, Israel and Libya, all of which are nesting sites belonging to the same cluster identified by Clusa et al. [111]. Therefore, it is expected that minimal nDNA differences are observed between most analysed specimens.

In contrast, specimens with CC-A1.1 and CC-A1.3 exhibited a stronger Atlantic character, as indicated by a lower contribution from cluster 1, while those with the Atlantic haplotype CC-A17.1 had an nDNA profile that more closely matched specimens from Haplogroup II (Figure 4). A similar pattern was also observed in DPCA analyses (Figure S2). This pattern may be explained by the existence of yet undiscovered Mediterranean rookeries with CC-A17.1 or by a closer genetic relationship between the Cape Verde population and that of the Mediterranean Sea. The absence of CC-A17.1 in other Mediterranean studies supports the latter scenario. None of the specimens in our study reached the 0.8 probability threshold suggested by Carreras et al. [28] for precise classification of Atlantic versus Mediterranean origin. Nonetheless, it is important to note that the current study utilised a different and larger set of microsatellite markers, which may have influenced the detection threshold.

### 4.3. Phylogeography

F_ST_ and φ_ST_ of this study highlight significant genetic differentiation between different foraging populations of *C. caretta*. The most significant *p*-values (<0.0001) were observed in the haplotype differences between the Atlantic and Mediterranean populations, indicating strong population structure and limited gene flow between these regions [22,62]. Within the Mediterranean, most pairwise comparisons also indicated significant divergence between populations, suggesting that different areas host distinct maternal lineages.

Notably, Mediterranean *C. caretta*, although maturing at a younger age than their Atlantic counterparts, still take more than 24 years to attain sexual maturity [71,72]. Despite these prolonged juvenile and sub-adult stages providing sufficient time for migration and mixing, the foraging populations remain genetically heterogeneous. In Turkey, Turkozan et al. [61] found differentiation in the origin of stranded *C. caretta* over a relatively small geographical scale. This variation likely reflects the nesting sites within the region and the migratory paths chosen, which may periodically shift according to the turtles’ life-cycle stages and are influenced by environmental factors [46,113].

### 4.4. Nests

In the current study, we identified the same haplotype, CC-A2.1, for the two analysed nests, which is the most common haplotype in the Mediterranean and Maltese nestlings [58]. To date, genetic analyses have confirmed that a total of eight females have used Maltese beaches for egg-laying (Table 6). We did not detect Atlantic haplotypes in the Maltese nests, and the addition of nuclear markers, along with cluster analyses, confirmed that all parents were of Mediterranean origin, as the hatchlings’ nDNA profile closely matched that of most foraging specimens around Malta. Microsatellite analyses of nests in Spain and Lampedusa have genetically attributed some nests to Atlantic or mixed-origin parents [28], suggesting that specimens of Atlantic origin occasionally use western and central Mediterranean beaches for nesting as well.

### 4.5. Conservation Value

Wallace et al. [34] documented multiple RMUs for *C. caretta*, designating the Mediterranean Sea as a distinct RMU, which is also frequented by specimens from the northwest and northeast Atlantic RMUs [21,22,23,34,112]. Within the Mediterranean RMU, based on genetic data from rookeries, Shamblin et al. [49] identified seven MUs, with the area studied here being in close proximity to the Libyan and Italian MUs. Despite its importance as a foraging ground and a migratory corridor [46,113], *C. caretta* populations in the central Mediterranean face several threats from intense human activity, including by-catch, coastal development and marine traffic [2,114], emphasising the need for targeted conservation measures.

Additionally, the Sicilian Channel, which serves as a transition zone between the warmer eastern basin and cooler western basin, is a key area for monitoring genetic shifts and understanding changes in migratory patterns. Recent records of increased sporadic loggerhead nesting in Malta and on several western Mediterranean beaches suggest possible shifts in distribution and new colonisations from the eastern Mediterranean and Atlantic rookeries [28,40,41,42]. This study highlights the central Mediterranean’s role as a foraging ground for turtles from various rookeries. Understanding connectivity between foraging areas and nesting sites, as well as migratory routes, is essential for conservation management.

To support these efforts, routine genetic sampling is essential for building a comprehensive time series, enabling the detection of genetic changes tied to management practices, human activity, and environmental shifts such as climate change. Such data will aid in protecting not only local populations but also migratory specimens. Furthermore, Maltese beaches are also nesting sites for *C. caretta*, with annual nests being recorded in recent years. This trend highlights the growing importance of Maltese water’s role in this species’ reproductive biology as well. Implementing genetic monitoring for these nesting turtles offers insights into the genetic diversity of the species, allowing for the early detection of changes. This information will enhance ongoing conservation efforts, ensuring that strategies are effective in preserving this flagship species for future generations [58].

## 5. Conclusions

Our research, analysing mtDNA and nDNA from 63 specimens collected in Maltese waters, highlighted the genetic structure of *C. caretta* in the region and confirmed the presence of individuals originating from multiple rookeries. Identifying the contributing rookeries is important in understanding links between nesting sites and foraging grounds. Notably, the detection of the Atlantic haplotype CC-A17.1 represents the first record of this haplotype in the region. The observed increase in genetic diversity over time may be indicative of an increasing number of migrants, possibly from both the Atlantic, as seen from the mtDNA data, and from other Mediterranean regions. This knowledge, combined with the increasing number of females using Maltese beaches for nesting [58], is essential for future monitoring and conservation programmes.

The genetic input from various nesting sites, including Atlantic populations, and the observed differentiation from other Mediterranean foraging regions emphasises the need for protecting a diverse range of habitats to support the species’ survival and its genetic diversity. Additionally, the central Mediterranean can serve as a valuable site for monitoring genetic variation over time, facilitating the detection of population changes in response to conservation efforts and environmental shifts.

## Figures and Tables

**Table 1:** The number of specimens per haplotype identified during this study for each sampling period, including the associated rookeries for each haplotype. The table includes the haplotype diversity (*h* ± SD), nucleotide diversity (*π* ± SD) and mean pairwise difference (±SD) between samples between different sampling periods.

Haplotype	2008–2009 (n = 14)	2014–2018 (n = 15)	2022–2023 (n = 34)	Total	Rookeries ^1^
CC-A1.1			1	1	Med: see note ^2^ Atl: GEO, FLO1, FLO2, CSL, DRT, MEX, KEY, CSK, FLO3
CC-A1.3			3	3	Med: not detected Atl: FLO1, FLO2, DRT, MEX, CSK, CAP
CC-A17.1		1	1	2	Med: not detected Atl: CAP
CC-A2.1	12	10	21	43	Med: CAL, GRE, CRT, DLY, DAL, TKW, TME, CYP, LIR, LYB Atl: FLO1, FLO2, CSL, DRT, MEX, KEY, CSK, FLO3, CAP
CC-A2.9		2	4	6	Med: LIR, LYB Atl: not detected
CC-3.1	2		2	4	Med: DLY, DAL, TKW, TME, LIR, LYB Atl: FLO1, FLO2, CSL, MEX, KEY, CSK, FLO3
CC-A6.1			1	1	Med: GRE Atl: not detected
CC-A26.1			1	1	Med: LYB Atl: not detected
CC-A29.1		1		1	Med: LIR, TKW Atl: not detected
CC-A53.1		1		1	Med: TME Atl: not detected
*h*	0.264 ± 0.136	0.562 ± 0.143	0.608 ± 0.092	0.525 ±0.074	
*π*	0.0003 ± 0.0004	0.0072 ± 0.0041	0.0129 ± 0.0067	0.0090 ±0.0047	
pairwise	0.26 ± 0.31	6.21 ± 3.13	11.11 ± 5.17	7.71 ±3.64	

^1^ Shamblin et al. [21].; Clusa et al. [35]; Carreras et al. [28]; Kaska et al. [106]; Luna-Ortiz et al. [33]. ^2^ Haplotype has been detected in sporadic nests from the western Mediterranean [28,33]. Abbreviations of rookeries according to Loisier et al. [23]: GEO (Cape Island, South Carolina + Ossabaw Island, Georgia), FLO1 (Canaveral National Seashore + Melbourne Beach, Florida), FLO2 (Juno Beach + Ft. Lauderdale, Florida), CSL (Cay Sal, Bahamas), DRT (Dry Tortugas, Florida), MEX (Isla Cozumel + Quintana Roo mainland, Mexico), KEY (Keewaydin Island, Florida), CSK (Casey Key, Florida), FLO3 (St. George Island + Cape San Blas, Florida), BRA (Sergipe + Bahia + Espírito Santo + Rio de Janeiro, Brazil), CAP (Boa Vista + Sal + Santa Luzia + Maio, Cape Verde), CAL (Calabria, Italy), GRE (Zakynthos Island + Kyparissia + Lakonikos, Greece), CRT (Rethymno, Crete), DLY (Dalyan, Turkey), DAL (Dalaman, Turkey), TKW (western Turkey), TME (middle Turkey + eastern Turkey), CYP (Alagadi + Akamas, Cyprus), LIR (El Mansouri, Lebanon + Israel), LYB (Sirte + Misurata, Lybia).

**Table 2:** Mitochondrial DNA pairwise F_ST_ values above the diagonal and φ_ST_ below the diagonal for the various sampling periods. Microsatellite pairwise F_ST_ values for the various sampling periods.

	mtDNA			Microsatellites		
	2008–2009	2014–2018	2022–2023	2008–2009	2014–2018	2022–2023
	(n = 14)	(n = 15)	(n = 34)	(n = 13)	(n = 15)	(n = 33)
**2008–2009**	-	0.0665	0.0094	-		
**2014–2018**	0.0359	-	<0.0001	0.0109	-	
**2022–2023**	0.0347	<0.0001	-	0.0040	<0.0001	-

None of these showed significant differentiation at the 95% confidence level following Bonferroni correction.

**Table 3:** Microsatellite data per locus. n = number of specimens analysed; N_a_ = number of alleles detected; H_o_ = observed heterozygosity; H_E_ = expected heterozygosity; P_HWE_ = probability of genotype proportions in conformance with expectations of the Hardy–Weinberg equilibrium. PIC = polymorphic informative loci.

*tetra*	Cc1G02	Cc1G03	Cc2H12	Cc5H07	Cc7B07	Cc7E11	Cc7G11	Cc8E07	CcP1F09	CcP5C11	CcP7D04	CcP7F06	CcP7H10	Mean Tetra
**n**	61	61	61	61	61	61	59	61	59	61	61	61	60	60.62
**N_a_**	15	15	14	14	16	13	9	13	12	4	14	13	6	12.15
**H_O_**	0.852	0.852	0.836	0.967	0.869	0.787	0.797	0.918	0.847	0.492	0.852	0.836	0.683	0.814
**H_E_**	0.909	0.898	0.860	0.897	0.904	0.839	0.848	0.900	0.851	0.595	0.900	0.843	0.707	0.842
**P_HWE_**	0.806	0.365	0.570	0.103	0.385	0.648	0.855	0.400	0.027	0.093	0.645	0.951	0.475	0.564
**PIC**	0.894	0.881	0.838	0.880	0.887	0.816	0.823	0.884	0.825	0.591	0.884	0.819	0.657	0.821
***di***	**cc7**	**cc117**	**cc141**	**Ccar176**	**Cc-2**	**Cc-8**	**Cc-10**	**Cc-17**	**Cc-22**	**Cc-25**	**Cc-28**	**Cc-30**	**Mean** **di**	**Mean** **Overall**
**n**	61	61	60	60	61	61	60	58	61	60	61	59	60.25	60.44
**N_a_**	13	10	10	11	5	7	5	10	8	7	5	9	8.33	10.32
**H_O_**	0.918	0.770	0.783	0.617	0.426	0.508	0.650	0.534	0.574	0.800	0.721	0.644	0.662	0.741
**H_E_**	0.889	0.811	0.792	0.626	0.436	0.599	0.713	0.629	0.640	0.764	0.725	0.772	0.670	0.774
**P_HWE_**	0.712	0.861	0.044	0.624	0.452	0.456	0.375	0.013	0.200	0.636	0.037	0.047	0.536	0.554
**PIC**	0.871	0.779	0.762	0.587	0.407	0.534	0.658	0.593	0.604	0.722	0.669	0.737	0.660	0.744

No significant deviations from HWE after using Bonferroni correction.

**Table 4:** AMOVA analyses of microsatellite data using haplotypes as prior for grouping. The grouping of haplotypes corresponds to that shown in Figure 4.

	Percentage Variation			
Groups	Among Groups	Among Populations Within Groups	Within Populations	F_ST_
**Haplogroup IB vs. Haplogroup II**	3.59	0.31	96.10	0.039 (*p* = 0.0123)
**CC-A1.1 + CC-A1.3 vs. the rest**	5.19	0.29	94.52	0.055 (*p* = 0.0126)
**CC-A1.3 vs. the rest**	6.08	0.40	93.52	0.061 (*p* = 0.0122)

**Table 5:** The pairwise F_ST_ values (above diagonal) and φ_ST_ values (below diagonal) for several foraging populations of *Caretta caretta*.

	Malta (n = 63) Current Study	Turkey (n = 135) [61]	Adriatic Sea (n = 488) [38]	French Coast (n = 158) [23]	North Atlantic (n = 388) [51]
**Malta**	-	0.1275 **	0.2183 **	0.0169	0.2398 **
**Turkey**	0.0465 *	-	0.0098 *	0.0317 **	0.3809 **
**Adriatic Sea**	0.0440 **	0.0077	-	0.0475 **	0.4994 **
**French coast**	0.0219 *	0.0045	0.00023	-	0.3316 **
**North Atlantic**	0.1700 **	0.2614 **	0.3048 **	0.2301 **	-

* Significant difference with *p*-values < 0.05. ** Significant difference with *p*-values < 0.005 (after Bonferroni correction).

**Table 6:** A list of genetically analysed nests from the Maltese islands. Each nest is given a Nest Reference Code, and the table includes the nesting year, the number of dead specimens analysed and the mtDNA haplotype associated with the nest. The Mother and Father codes are assigned to the parents associated with each nest based on microsatellite data analyses.

Nesting Site	Nesting Year	Nest Reference Code	Specimens Analysed	mtDNA Haplotype	Mother Code	Father Code	Ref.
Fajtata Bay	2020	CFA	23	CC-A2.1	1	1	^a^
Għadira Bay	2020	CGA	44	CC-A2.1	2	2	^a^
Għadira Bay	2020	CGB	7	CC-A2.1	2	2	^a^
Ramla Bay	2020	CRA	1	CC-A2.1	3	3	^a^
Golden Bay	2020	CMA	5	CC-A2.1	3	3	^a^
Ramla Bay	2020	CRB	7	CC-A2.1	4	4	^a^
Ramla Bay	2021	CRC	7	CC-A2.1	5	5	^a^
Ramla Bay	2022	CRD	26	CC-A3.1	6	6 + 7	^a^
Ramla Bay	2023	CRE	6	CC-A2.1	7	8	^b^
Ġnejna Bay	2023	CNA	1	CC-A2.1	8	9	^b^

^a^ Vella and Vella [58]. ^b^ current study.

## Data Availability

Haplotype names used follow the Archie Carr Center for Sea Turtle Research database.

## Supplementary Materials

The following supporting information can be downloaded at https://www.mdpi.com/article/10.3390/genes15121565/s1, Figure S1: Haplotype rarefaction curve for the Maltese samples, including the 95% confidence intervals in grey; Figure S2: A plot showing the discriminant function vs. density for the two groupings as identified through STRUCTURE; Table S1: The values obtained for the various data sets as run through BOTTLENECK. Data includes p-values from bottleneck tests using three different mutation models: the infinite allele model (IAM), the two-phase model (TPM: 10% IAM and 90% SMM), and the stepwise mutation model (SMM); Table S2: The genetic data per nest, including the mtDNA haplotypes, the sample sizes per locus (n), the number of alleles identified per locus (N_a_), and the observed heterozygosity per locus (H_o_).
