# Supplementary material for: Genetic Identity and Diversity of Loggerhead Sea Turtles in the Central Mediterranean Sea
Source: Genes (Basel). 2024 Dec 2;15(12):1565. doi: 10.3390/genes15121565 (PMC11728243; doi:10.3390/genes15121565)
Supplement: Supplementary file 1 [file genes-15-01565-s001.zip › genes-3343246-supplementary.pdf]

Supplementary material:

Figure S1: Haplotype rarefaction curve for the Maltese samples, including the 95% confidence intervals in grey.

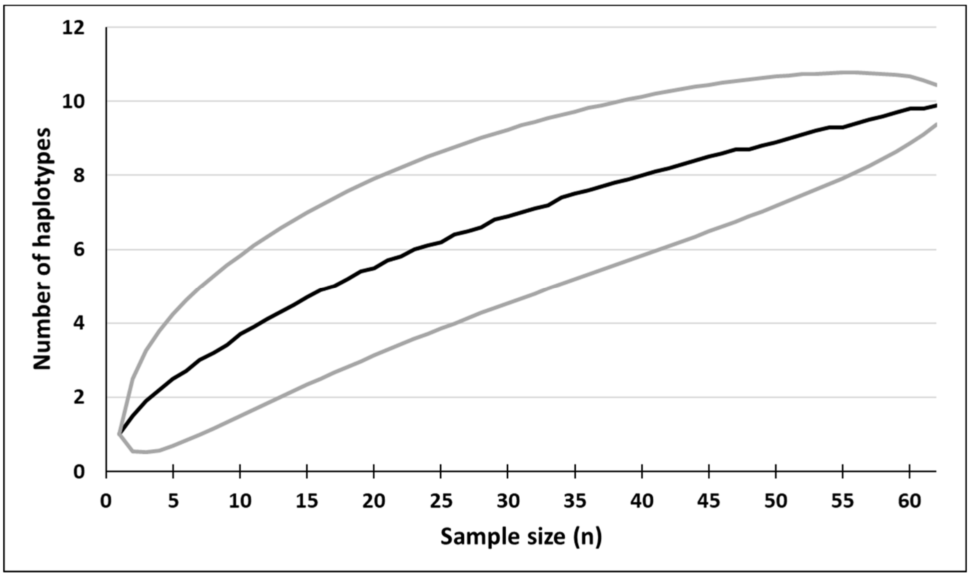

Figure S2: A plot showing the discriminant function vs density for the two groupings as identified through STRUCTURE.

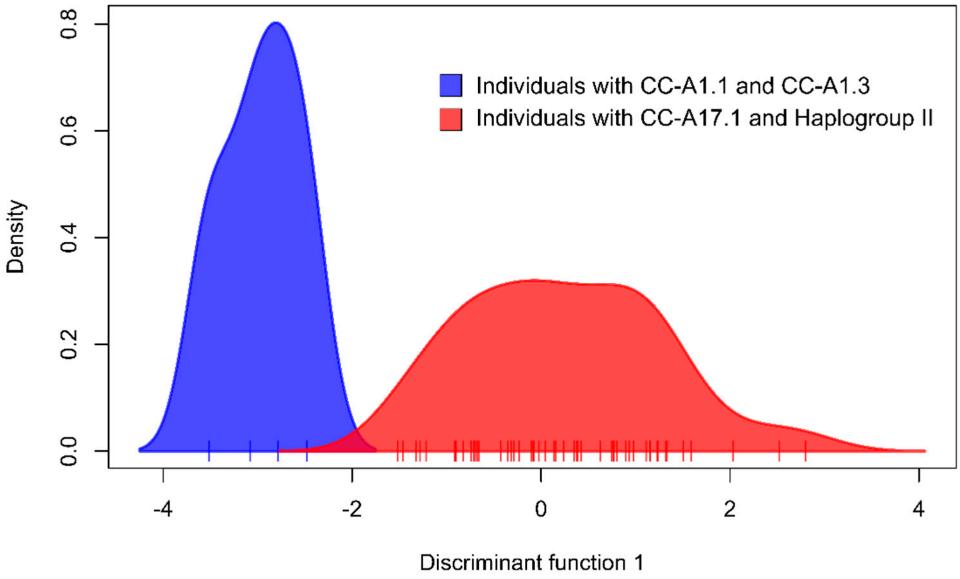

Table S1: The values obtained for the various data sets as run through BOTTLENECK. Data includes *p*-values from bottleneck tests using three different mutation models: the infinite allele model (IAM), the two-phase model (TPM: 10% IAM and 90% SMM), and the stepwise mutation model (SMM).

**Population**	**Test**	**IAM**	**TPM**	**SMM**
**All sample**	Sign test: expected number of loci with heterozygosity excess (probability)	14.09 (0.0001)	14.75 (0.5449)	14.81 (0.0897)
	Standardised differences test: T2 values (probability)	4.485 (0.0000)	0.745 (0.2282)	-1.346 (0.0891)
	Wilcoxon test: probability of heterozygote excess	0.0000	0.2054	0.8237
**2008 – 2009**	Sign test: expected number of loci with heterozygosity excess (probability)	14.04 (0.0007)	14.63 (0.3657)	14.76 (0.5476)
	Standardised differences test: T2 values (probability)	3.518 (0.0002)	1.116 (0.1323)	0.097 (0.4615)
	Wilcoxon test: probability of heterozygote excess	0.0000	0.0705	0.3360
**2014 – 2018**	Sign test: expected number of loci with heterozygosity excess (probability)	14.12 (0.0124)	14.78 (0.2449)	14.76 (0.5471)
	Standardised differences test: T2 values (probability)	3.425 (0.0003)	0.832 (0.2027)	-0.308 (0.3792)
	Wilcoxon test: probability of heterozygote excess	0.0001	0.2209	0.4895
**2022 – 2024**	Sign test: expected number of loci with heterozygosity excess (probability)	13.78 (< 0.0001)	14.76 (0.2412)	14.77 (0.4518)
	Standardised differences test: T2 values (probability)	4.709 (< 0.0001)	1.759 (0.0393)	0.313 (0.3772)
	Wilcoxon test: probability of heterozygote excess	< 0.0001	0.0226	0.2131
**Haplogroup II**	Sign test: expected number of loci with heterozygosity excess (probability)	14.05 (0.0007)	14.86 (0.2543)	14.88 (0.1655)
	Standardised differences test: T2 values (probability)	4.503 (< 0.0001)	0.959 (0.1687)	-0.935 (0.1750)
	Wilcoxon test: probability of heterozygote excess	< 0.0001	0.1050	0.7199

Table S2: The genetic data per nest, including the mtDNA haplotypes, the sample sizes per locus (n), the number of alleles identified per locus (N_a), and the observed heterozygosity per locus (H_o).

	CNA	CRE	overall
mtDNA	CC-A2.1	CC-A2.1	
**Cc-30**			
n	1	6	120
N_a	2	2	5
H_o	1.000	0.500	0.867
**Cc2H12**			
n	1	6	117
N_a	2	4	9
H_o	1.000	1.000	0.667
**Cc-22**			
n	1	6	120
N_a	2	2	7
H_o	1.000	1.000	0.725
**Cc-28**			
n	1	6	121
N_a	2	2	4
H_o	1.000	0.667	0.686
**Cc-17**			
n	1	6	116
N_a	2	2	4
H_o	1.000	1.000	0.595
**Cc-8**			
n	1	6	121
N_a	2	2	5
H_o	1.000	0.167	0.636
**Cc-2**			
n	1	6	119
N_a	2	2	4
H_o	1.000	0.500	0.479
**cc7**			
n	1	6	120
Na	2	2	9
Ho	1.000	0.333	0.917
**Ccar176**			
n	1	6	120
N_a	2	2	6
H_o	1.000	0.833	0.608
**Cc7G11**			
n	1	6	116
N_a	2	4	8
H_o	1.000	1.000	0.638
**cc141**			
n	1	6	120
N_a	2	3	6
H_o	1.000	0.833	0.633
**Cc1G02**			
n	1	6	120
N_a	2	3	12
H_o	1.000	1.000	0.808
**CcP1F09**			
n	1	6	112
N_a	2	4	9

H_o	1.000	1.000	0.813
**Cc5H07**			
n	1	6	115
N_a	2	3	11
H_o	1.000	1.000	0.948
**CcP7D04**			
n	1	6	116
N_a	2	4	13
H_o	1.000	1.000	0.922
**CcP5C11**			
n	1	6	119
N_a	2	3	4
H_o	1.000	0.833	0.555
**CcP7F06**			
n	1	6	116
N_a	2	3	9
H_o	1.000	0.667	0.983
**Cc-10**			
n	1	6	118
N_a	2	3	4
H_o	1.000	1.000	0.898
**Cc7B07**			
n	1	6	121
N_a	2	4	10
H_o	1.000	0.833	0.959
**Cc7E11**			
n	1	6	118
N_a	2	3	9
H_o	1.000	0.833	0.720
**cc117**			
n	1	6	119
N_a	2	2	7
H_o	1.000	1.000	0.647
**Cc-25**			
n	1	6	119
N_a	2	3	6
H_o	1.000	1.000	0.647
**CcP7H10**			
n	1	6	120
N_a	2	3	5
H_o	1.000	0.833	0.658
**Cc8E07**			
n	1	6	119
N_a	2	4	13
H_o	1.000	1.000	0.941
**Cc1G03**			
n	1	6	121
N_a	2	3	10
H_o	1.000	0.705	0.752
